# Psychedelic Psychiatry

**DOI:** 10.1192/j.eurpsy.2022.36

**Published:** 2022-09-01

**Authors:** D. Nutt

**Affiliations:** Imperial College London, Psychiatry, London, United Kingdom

## Abstract

The last decade has seen a remarkable resurgence of interest in psychedelic drugs such as psilocybin (from magic mushrooms) LSD and DMT (dimethyl tryptamine – the active ingredient of ayahuasca). This has been driven by the discovery that these psychedelics all act agonists of 5-HT2A receptors. Human imaging studies have revealed this action leads to profound alterations in brain measures of activity particularly in terms of increased entropy of EEG MEG and fMRI signals and reduced within-network, but increased between-network, connectivity. In addition they all can increase synaptic growth and brain plasticity. These findings not only explain the subjective nature of the psychedelic experience but also have implications for the treatment of internalising disorders such as depression addiction anorexia and OCD that are characterised by increased within network connectivity especially of the default mode network. Subsequent trials, particularly of psilocybin, in these disorders has revealed significant clinical benefits from even just a single administration. A number of companies have now been set up to extend these discoveries with regulatory-level trials that could result in market authorisations within a few years. My talk will explore these brain mechanisms and clinical data and discuss the potential place of psychedelic medicine in the future of psychiatry.

**Disclosure:**

I am an advisor to Compass pathways and Beckley Psytec two companies that are developing psychedelics for depression and other psychiatric indications. Several members of my research group receive support from these companies and also from Small Pharma.

